# An Ontology-Powered Dialogue Engine For Patient Communication of Vaccines

**Published:** 2019-10

**Authors:** Muhammad Amith, Rebecca Lin, Licong Cui, Dennis Wang, Anna Zhu, Grace Xiong, Hua Xu, Kirk Roberts, Cui Tao

**Affiliations:** 1 The University of Texas Health Science Center at Houston, Houston, TX 77030; 2 Johns Hopkins University, Baltimore, MD; 3 The University of Texas, Austin, TX; 4 Southern Methodist University, Dallas, TX

**Keywords:** Ontology, Dialogue Management, Question Answering, Vaccines, Conversational Agent

## Abstract

In this study, we introduce an ontology-driven software engine to provide dialogue interaction functionality for a conversational agent for HPV vaccine counseling. Currently, the HPV vaccination rates are low that risks unprotected individuals at being infected with HPV, a virus that leads to life-threatening cancers. In addition, we developed a question answering subsystem to support the dialogue engine. In this paper, we discuss our design and development of an ontology-driven dialogue engine that uses the Patient Health Information Dialogue Ontology, an ontology that we previously developed, and a question answering subsystem based on various previous methods to supplement the dialogue engine’s interaction with the user. Our next step is to test the functional ability of the ontology-driven software components and deploy the engine in a live environment to be integrated with a speech interface.

## 1 Introduction

Speech is the most natural and effective way for us to communicate. Through speech, we can communicate a lot of information in very little time compared to printed material [16,6,13]. Face-to-face communication between a health provider and patient is an important factor in improving the health outcome of consumers. This is particularly beneficial in patient-provider communication for the human papillomavirus (HPV) vaccine, an effective vaccine that prevents adulthood cancers. Research has shown that provider communication could potentially increase the vaccine uptake substantially [10]. In addition, the President’s Cancer Council recommends provider communication to improve uptake rates [18]. However, HPV vaccine rates are below the 80% target coverage rate [20]. This is compounded with the presumption that health providers deal with compressed time to thoroughly discuss the HPV vaccine and answer their questions, and only a third of patients partake in discussion for the HPV vaccine [10]. A dialogue system is a computer-based agent that converses with human users using either text or speech. Our experimental proposition is a speech-enabled dialogue system embodied in a software agent that could inhabit a clinical environment. This agent could administer the communication task of counseling patients on the HPV vaccine.

**Fig. 1.**
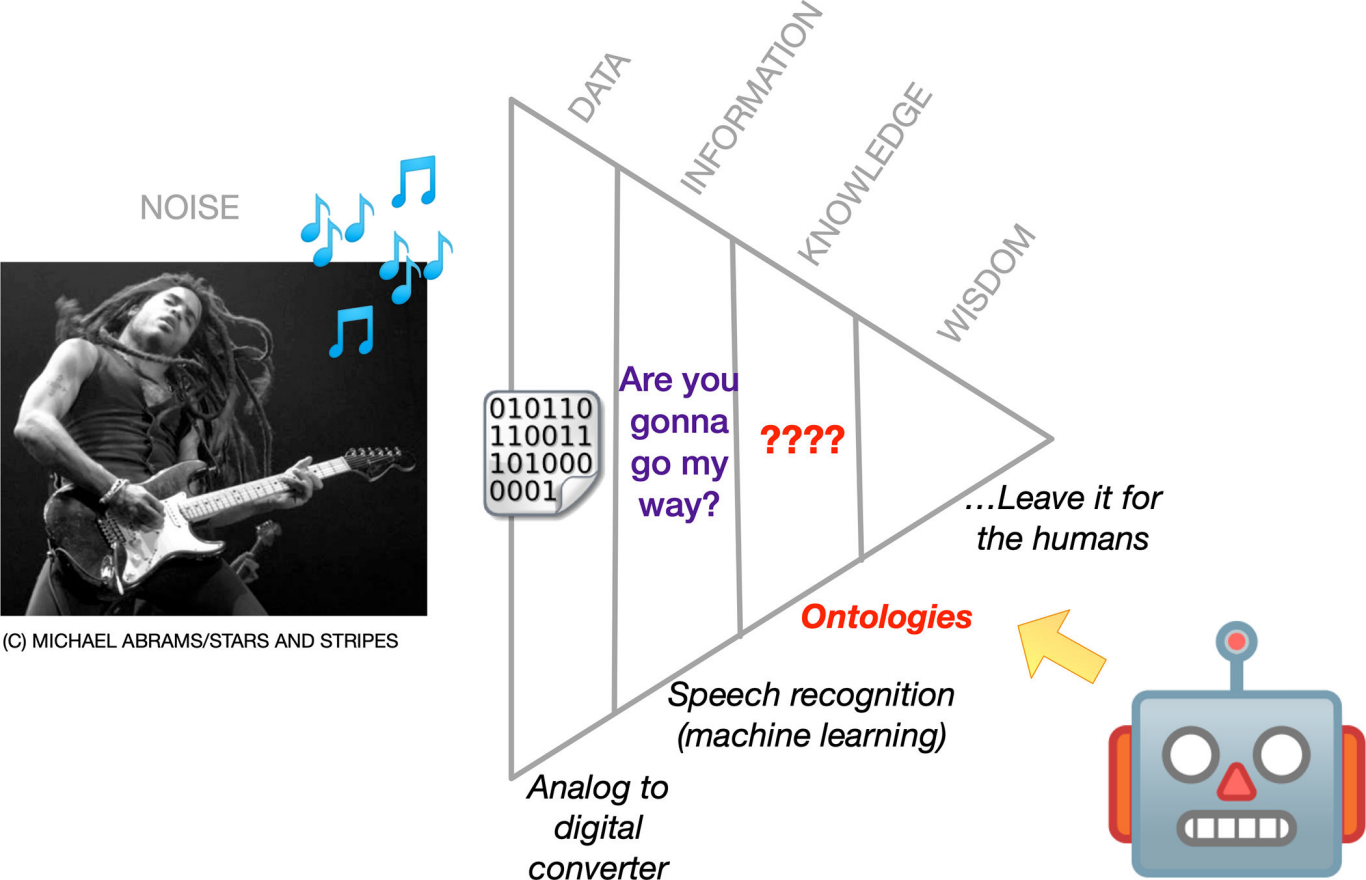
Application of the knowledge pyramid for agents.

Aside from being expressive terminologies in the biomedical field, ontologies can provide support for autonomous software agents [12], akin to Tim BernersLee’s vision for ontologies in agents [4]. Take the classic knowledge pyramid, applied in an agent-based use case (Figure 1). An artist playing music emits audio noise (*Noise*) that can be translated into digital format by a robot’s analog-to-digital converter (*Data*). The digital data can be further processed by the machine’s speech recognition software to convert the digital data to information - string text (*Information*). However, the machine needs to know the rules on how to react and behave when presented with information (*Knowledge*). Within this example, ontologies occupy a unique role for software agents in the evolution of information on the knowledge pyramid [9].

We discuss our prototype software engine, named the Conversational Ontology Operator (COO), that utilizes an ontology for dialogue management to coordinate the conversational behavior. This engine is a prototype that we plan to integrate into an embodied agent to provide the autonomous interaction intelligence to discuss health information with a patient. This engine will not only coordinate the dialogue exchanges with the user, but also answer vaccine questions from the user. This task is facilitated by a question answering subsystem for ontologies that we call Frankenstein ^5^ Ontology Question Answering for User Centric Systems (FOQUS). This QA system will utilize our previously developed VISO-HPV [21] as a knowledge base to query.

## 2 Materials and Method

We first collected data from a Wizard of OZ experiment [8], and that data led to the development of the ontology-driven dialogue system engine. The following subsections describes our development endeavor.

### Data collection

The genesis of this work began with simulation studies involving potential participants (*n* =16) who fit the demographic that the conversational agent (CA) is targeted for - parents with at least one child under 18 [2]. The simulation was a Wizard of OZ experiment that mimicked the CA using an iPad tablet and a desktop application that transmitted an operators’ utterance to the tablet, while masquerading as an autonomous CA. We used the dialogue exchanges recorded in a chat log and the dialogue script to analyze unique interaction patterns and parse out participant questions (53 in total). The utterances and interaction patterns helped us generate an application ontology for CA for health - Patient Health Information Dialogue Ontology (PHIDO).

### Patient Health Information Dialogue Ontology

We produced an ontology for health dialogue management, called PHIDO [1], that can facilitate dialogue flow and contextual dialogue information for a software agent. PHIDO provides the concepts to create a framework of health counseling between human and machine. We used PHIDO to create a reusable model of a HPV vaccine counseling encounter. PHIDO describes the various utterance and speech task classes, and their object and data property links, to coordinate the dialogue. This model contains three basic speech tasks that can be linked together to form a discussion. This ontology can later be integrated with health intervention models to build upon and validate these models.

### Conversational Ontology Operator

**Fig. 2.**
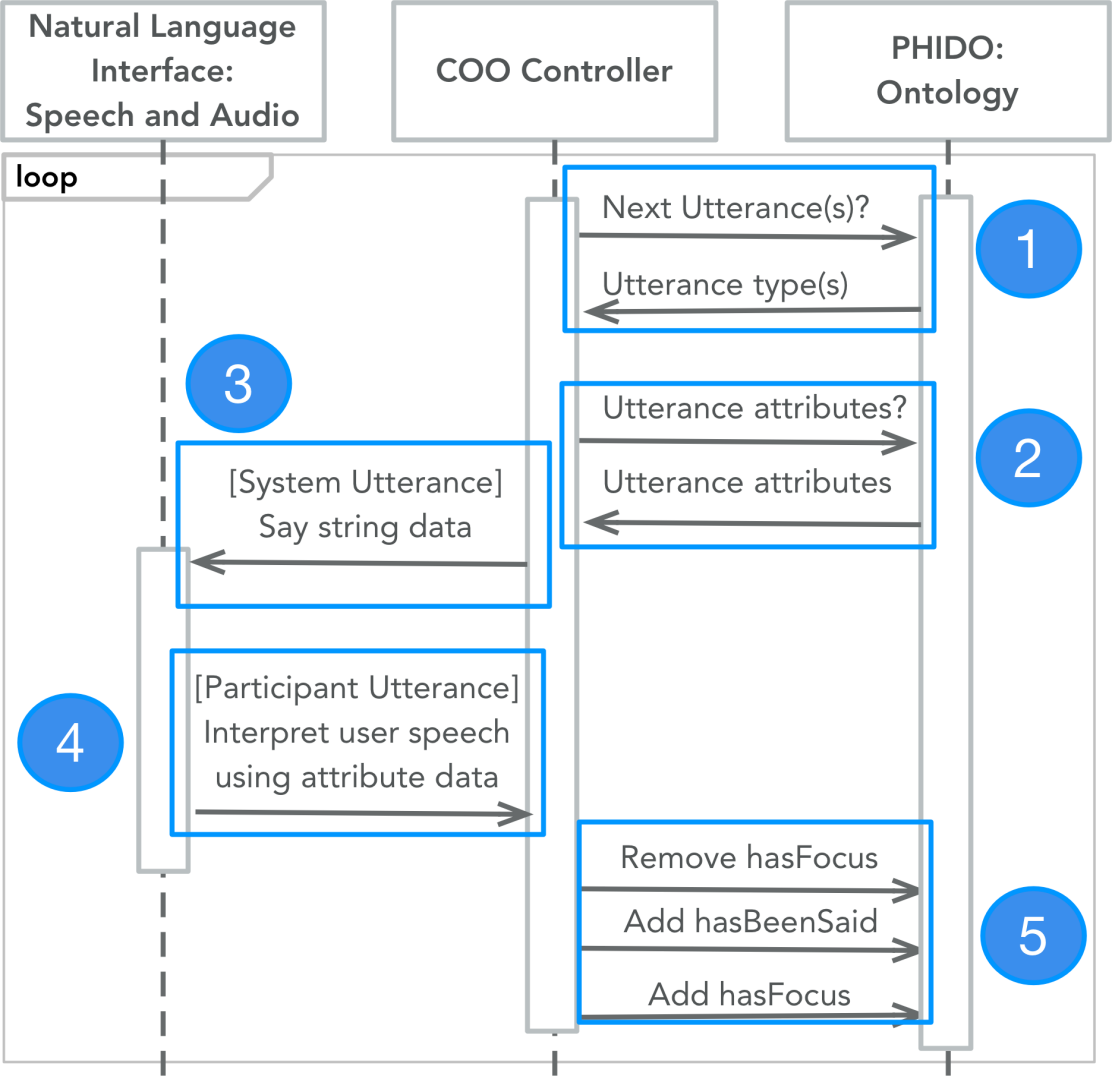
UML sequence diagram outlining the dialogue transition sequence implemented in the COO engine.

Our previous steps culminated in the development of Conversational Ontology Operator (COO), a software engine that manages the dialogue for the agent. COO implements a transition mechanism coordinated by PHIDO (Figure 2). To summarize, COO implements a continuous loop where it first queries for the current position of the dialogue based on a data property (*hasFocus*) (*Part 1* of Figure 2). Afterwards, it queries for the next utterance instances and collects their data (*2* of Figure 2). If the utterance instance is a system-related utterance, the agent will communicate with the participant (*3* of Figure 2), or if it is a participant utterance it will determine what type of utterance the user spoke (i.e. using the data associated with the utterance instance) (*4* of Figure 2). Lastly, COO will update the position of the dialogue (*hasFocus*) and repeat (*5* of Figure 2).

### Frankenstein Question Answering for User-Centric Systems

**Fig. 3.**
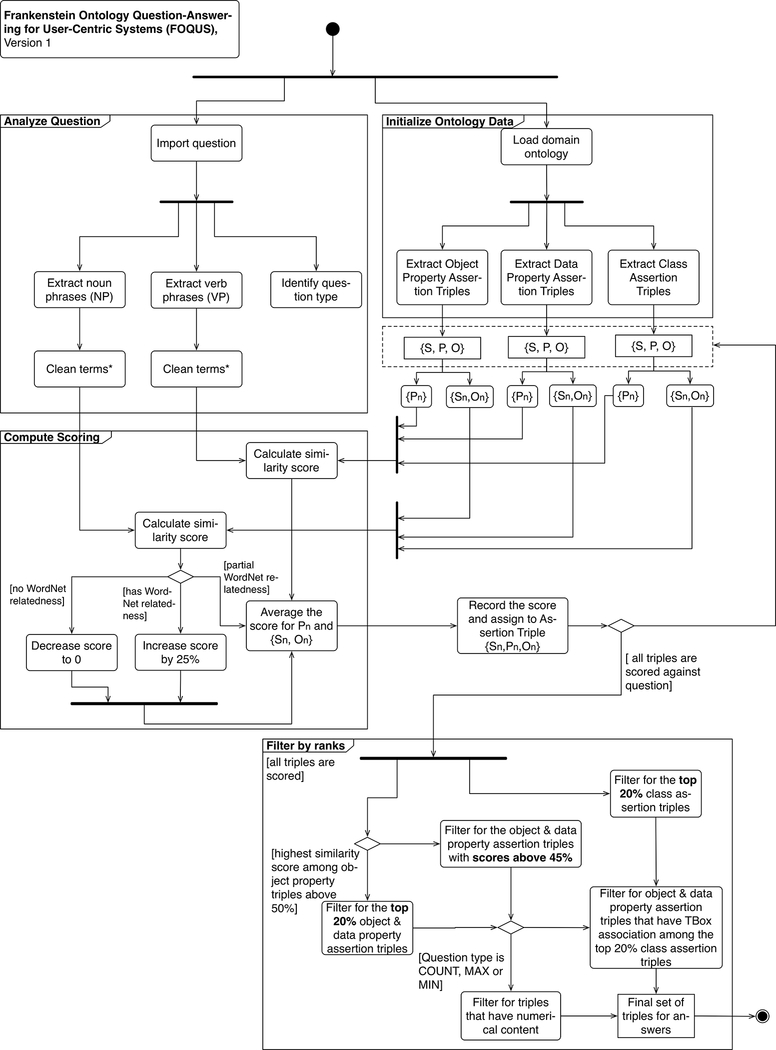
UML activity diagram describing FOQUS’ process implementation.

As an accessory with COO, we co-cleveloped a subsystem for question answering (QA) to respond to consumer questions during the automated counseling. Using a domain ontology, this QA subsystem called Frankenstein Question Answering for User-Centric Systems (FOQUS) will query an answer from a natural language question expressed by the user. The question’s noun phrases (NP) and verb phrases (VP) are extracted, including its question type determined by keyword-based identification. The domain ontology’s Object Property, Data Property, and Class Assertions are parsed and then compared with the NP and VP for similarity. A score is assigned for each assertion axiom (triple). Various rules are applied to find the top ranking triples, and from those selected triples, we compose a natural language response using simple rules for aggregating and compounding triples. See Figure 3 of the Appendix which outlines the question answering method.

## 3 Discussion

COO and FOQUS were developed using Java 8, with Eclipse RDF4j [7], OWLAPI [14], extJWNL [3], Stanford CoreNLP [17] and HermiT reasoning [11] libraries. For FOQUS, similarity methods employed string-based matching from SimMetrics [15] and vector-based comparisons using Numberbatch [19].

Our next endeavor with this project is to test the functionality of both the dialogue engine and the question answering system. Most of the dialogue interaction is primarily communicating the singular pieces of information about the HPV and HPV vaccine. We will focus on the core dialogue exchange which is the communication of health information to the user as our test example. To assess the COO engine’s interaction, we will observe if the system can impart a piece of health information (HPV vaccine-related) to the user, coordinate question answering, and transition the conversation to discuss a health topic.

To test FOQUS, we used questions asked during our simulated experiment with participants. In total, we collected 53 questions that range from age appropriateness for the vaccine, gender-related questions, cost, etc. Some of the questions may have been mis-transcribed from speech recognition, yet we kept it as is to imitate how the live system would process the question. Because of the possibility of mis-recognition of the utterances, FOQUS relies on the salient terms of the question (noun and verb phrases) to retrieve an answer. Enlisted evaluators will be asked to evaluate the question and answer pairs based on two criteria: *the acceptability of the answer for the questions* (on a 5 point Likert scale) and *whether the answer matches the question* (2=yes, 1=partial, 0=no). The first criterion aims to help us analyze the presentation and composition of the answer from triples. The second criterion helps us to determine if FOQUS can answer the question with some degree of relevancy. We calculated Cohen Kappa’s inter-rater reliability [5] for both of these criterion to determine the effectiveness of FOQUS.

## 4 Conclusion

We put forth an ontology-driven dialogue engine to provide an automated HPV vaccine counseling experience between a patient and a conversational agent. This paper presents our prototype ontology-driven dialogue engine (COO) with question-answering facilities (FOQUS). COO uses a previously developed dialogue ontology called PHIDO to direct and manage the interaction of the conversational agent for HPV vaccine counseling, and FOQUS uses our previously developed VISO-HPV to answer potential patient questions during the automated counseling experience. Our next step is to evaluate COO and FOQUS by demonstrating COO’s ability to fulfill functional use cases and FOQUS’s ability to answer sample questions from a simulated study. Our next goal is to deploy and test the software engine with potential users and assess its performance for possible use in a clinical environment.

## Footnotes

5 The inspiration behind the humorous name is due its patchwork of methods and ideas from various classic QA for ontologies (NLP-Reduce, FREyA, PANTO, etc.).
